# Loss of IDH2 Accelerates Age-related Hearing Loss in Male Mice

**DOI:** 10.1038/s41598-018-23436-w

**Published:** 2018-03-22

**Authors:** Karessa White, Mi-Jung Kim, Chul Han, Hyo-Jin Park, Dalian Ding, Kevin Boyd, Logan Walker, Paul Linser, Zaimary Meneses, Cole Slade, Jonathan Hirst, Katherine Santostefano, Naohiro Terada, Takuya Miyakawa, Masaru Tanokura, Richard Salvi, Shinichi Someya

**Affiliations:** 1 Department of Aging and Geriatric Research, University of Florida, Gainesville, Florida 32610 United States; 2 Center for Hearing and Deafness, State University of New York at Buffalo, New York, 14214 United States; 3 Whitney Laboratory, University of Florida, St Augustine, Florida 32080 United States; 4 Department of Pathology, Immunology and Laboratory Medicine, University of Florida, Gainesville, FL 32610 United States; 5 Department of Applied Biological Chemistry, University of Tokyo, Yayoi, Tokyo, 113 Japan

## Abstract

Isocitrate dehydrogenase (IDH) 2 participates in the TCA cycle and catalyzes the conversion of isocitrate to α-ketoglutarate and NADP^+^ to NADPH. In the mitochondria, IDH2 also plays a key role in protecting mitochondrial components from oxidative stress by supplying NADPH to both glutathione reductase (GSR) and thioredoxin reductase 2 (TXNRD2). Here, we report that loss of *Idh2* accelerates age-related hearing loss, the most common form of hearing impairment, in male mice. This was accompanied by increased oxidative DNA damage, increased apoptotic cell death, and profound loss of spiral ganglion neurons and hair cells in the cochlea of 24-month-old *Idh2*^−/−^ mice. In young male mice, loss of *Idh2* resulted in decreased NADPH redox state and decreased activity of TXNRD2 in the mitochondria of the inner ear. In HEI-OC1 mouse inner ear cell lines, knockdown of *Idh2* resulted in a decline in cell viability and mitochondrial oxygen consumption. This was accompanied by decreased NADPH redox state and decreased activity of TXNRD2 in the mitochondria of the HEI-OC1 cells. Therefore, IDH2 functions as the principal source of NADPH for the mitochondrial thioredoxin antioxidant defense and plays an essential role in protecting hair cells and neurons against oxidative stress in the cochlea of male mice.

## Introduction

In aerobic cells, the balance between antioxidant defenses and oxidants is determined by the ratios of inter-convertible forms of redox couples such as reduced glutathione (GSH)/oxidized glutathione (GSSG), reduced thioredoxin (TXN)/oxidized TXN, and NADPH (reduced nicotinamide adenine dinucleotide phosphate)/NADP^+^ (oxidized nicotinamide adenine dinucleotide phosphate)^1,2^. Of these redox couples, NADPH plays a crucial role in protecting cells from oxidative stress by serving as a co-factor for glutathione reductase (GSR) which reduces GSSG to GSH, and thioredoxin reductase (TXNRD) which reduces oxidized TXN to reduced TXN^3–6^. In the cytosol, NADPH is generated by glucose-6-phosphate dehydrogenase (G6PD), 6-phosphogluconate dehydrogenase (6PGD), isocitrate dehydrogenase 1 (IDH1) or malic enzyme 1 (ME1), while in the mitochondria, NADPH is generated from mitochondrial transhydrogenase (NNT), glutamate dehydrogenase (GLUD1), malic enzyme 3 (ME3) or IDH2^3,7,8^.

There are three isozymes of IDH: cytosolic IDH1, and mitochondrial IDH2 and IDH3. All isozymes catalyze the conversion of isocitrate to α-ketoglutarate. While IDH1 and IDH2 convert NADP^+^ to NADPH, IDH3 converts NAD^+^ to NADH^9,10^. Both IDH2 and IDH3 participate in the TCA (tricarboxylic acid) cycle in the mitochondrial matrix. Of the NADPH-producing enzymes in the mitochondria, IDH2 is thought to be a major source of NADPH for mitochondrial GSR and TXNRD2^9–11^. In support of this idea, overexpression of *Idh2* in NIH3T3 mouse fibroblasts increased cell survival and reduced levels of oxidative damage markers compared to control cells, while *Idh2*-knockdown resulted in decreased levels of mitochondrial NADPH and GSH, decreased cell survival, and increased markers of oxidative damage under H_2_O_2_ treatment^10^. In human cervical cancer cell lines, *Idh2*-knockdown resulted in increased levels of apoptosis markers, including DNA fragmentation, caspase-3, cytochrome *c*, and BAX^12^. In young mice, homozygous mutations in *Idh2* resulted in a 41% increase in heart size, extensive damage to the heart, and cardiac dysfunction^13^. Loss of *Idh2* also resulted in decreased levels of NADPH and increased oxidative damage markers in the kidney of young mice^14^. This was associated with greater kidney damage after ischemia-reperfusion compared to wild-type mice. In contrast, calorie restriction, known to extend lifespan in multiple species, increased NADPH levels and IDH2 activities in the mitochondria of the inner ear, brain, and liver of young mice^15^. Together, these reports suggest that IDH2 is a major source of NADPH for the mitochondrial antioxidant defenses under normal physiological conditions or stress conditions, while a loss of IDH2 results in increased oxidative stress, rendering cells vulnerable to oxidative DNA damage and apoptosis. In the current study, we show that loss of *Idh2* accelerated age-related hearing loss (AHL), the most common form of hearing impairment in humans^16^, in male mice. This was accompanied by increased oxidative DNA damage, apoptotic cell death, and profound loss of hair cells and spiral ganglion neurons in the cochlea. Biochemical analysis revealed that loss of *Idh2* resulted in decreased NADPH redox state and decreased activity of TXNRD2 in the mitochondria of inner ear tissues. In HEI-OC1 mouse inner ear cell lines, knockdown of *Idh2* resulted in a decline in cell viability and mitochondrial oxygen consumption. This was accompanied by decreased NADPH redox state and decreased activity of TXNRD2 in the mitochondria of the HEI-OC1 cells. Together, our findings provide evidence that IDH2 functions as the principal source of NADPH for the mitochondrial thioredoxin antioxidant defense and plays an essential role in protecting hair cells and neurons against oxidative stress in the cochlea of male mice.

## Results

### Backcrossing *Idh2* KO mice onto the CBA/CaJ mouse strain

**Figure 1:**
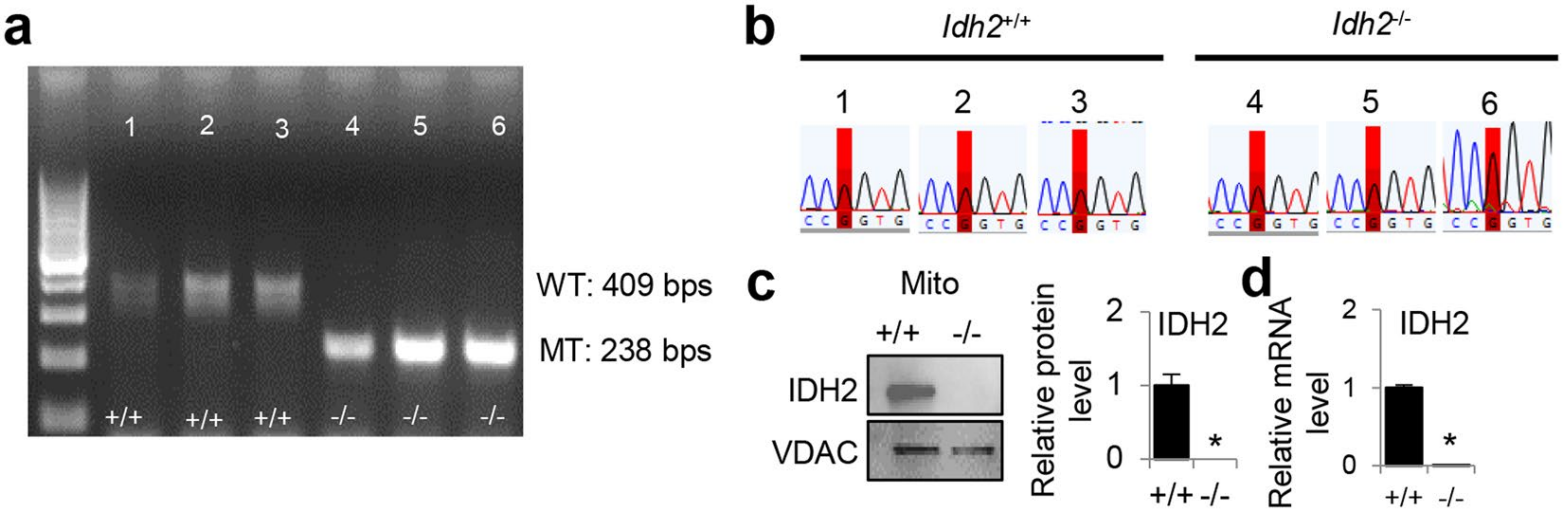
Genotyping of *Idh2*^+/+^*, Idh2*^+/−^, and *Idh2*^*−/−*^ mice. (**a)** PCR products were separated on a 1.5% agarose gel. The expected band sizes for the WT and mutant alleles were 409 bps and 238 bps, respectively. The full-length gel is presented in Supplementary Fig. 1. (**b**) The *Cdh23* gene in *Idh2*-deficient mice (n = 3 each) was sequenced. All the mice examined had the WT *Cdh23*^753G/753G^ genotype. Red blocks indicate the *Cdh23*^753G^ allele. (**c**) Western blotting analysis of IDH2 protein levels in the inner ear tissues from 5-month-old WT and *Idh2*^−/−^ males (n = 3–5). The full-length blot is presented in Supplementary Fig. 3. (**d**) *Idh2* mRNA relative expression levels in the inner ear tissues from 5-month-old WT and *Idh2*^−/−^ male mice (n = 3–5). Data are shown as means ± SEM. *p < 0.05 vs. WT. VDAC = Voltage-dependent anion channel.

To investigate the effects of *Idh2* deficiency on cochlear pathology and AHL in mice, *Idh2* heterozygous knockout mice were backcrossed for 5 generations onto the CBA/CaJ mouse strain, a well-established model of AHL^17,18^. We genotyped *Idh2*^+/+^ (WT) and *Idh2*^−/−^ mice by PCR genotyping (Fig. 1a and Supplementary Fig. 1) and then sequenced the *Cdh23* gene in the DNA obtained from tails of young mice. We confirmed that all WT and *Idh2*^−/−^ mice had the same WT *Cdh23* genotype (*Cdh23*^753G/753G^) (Fig. 1b). *Idh2*^+/−^ and *Idh2*^−/−^ mice on the CBA/CaJ background appeared phenotypically normal and viable. No significant changes in body weight were observed between WT, *Idh2*^+/−^ and *Idh2*^−/−^ males or female mice (Supplementary Fig. 2). To confirm the genotyping results, we measured the levels of *Idh2* protein and mRNA in inner ear tissues from 5-month-old WT and *Idh2*^−/−^ mice by Western blotting and quantitative RT-PCR. No IDH2 protein or little *Idh2* mRNA was detected in the inner ear of *Idh2*^−/−^ mice (Fig. 1c,d and Supplementary Fig. 3).

### Loss of *Idh2* accelerates age-related hearing loss in male mice

**Figure 2:**
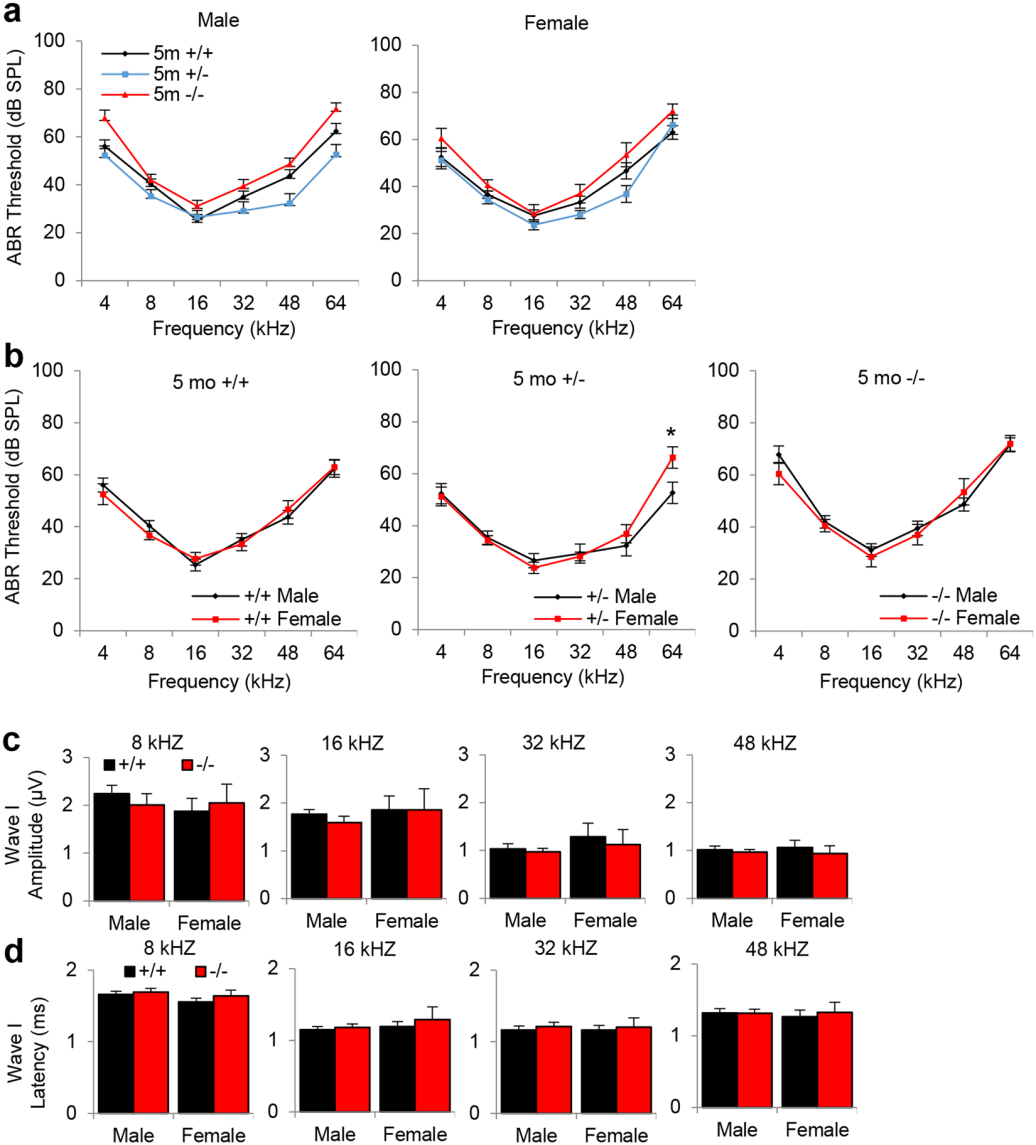
Assessment of ABR thresholds, amplitudes and latencies in young WT, *Idh2*^+/−^ and *Idh2*^−/−^ male and female mice. (**a**) ABR hearing thresholds were measured at 4, 8, 16, 32, 48 and 64 kHz in WT and *Idh2*^−/−^ male (left) and female (right) mice at 5 months of age (n = 10). (**b**) ABR hearing thresholds were measured at 4, 8, 16, 32, 48 and 64 kHz in WT (left), *Idh2*^+/−^ (middle), and *Idh2*^−/−^ (right) male and female mice at 5 months of age (n = 10). (**c,d**) ABR amplitudes (**c**) and latencies (**d**) of wave I were measured at 90 dB at 8, 16, and 32 kHz in 5-month-old WT and *Idh2*^−/−^ male and female mice (n = 10). Data are shown as means ± SEM. *p < 0.05 vs. young WT, *Idh2*^+/−^ or *Idh2*^−/−^ mice.

**Figure 3:**
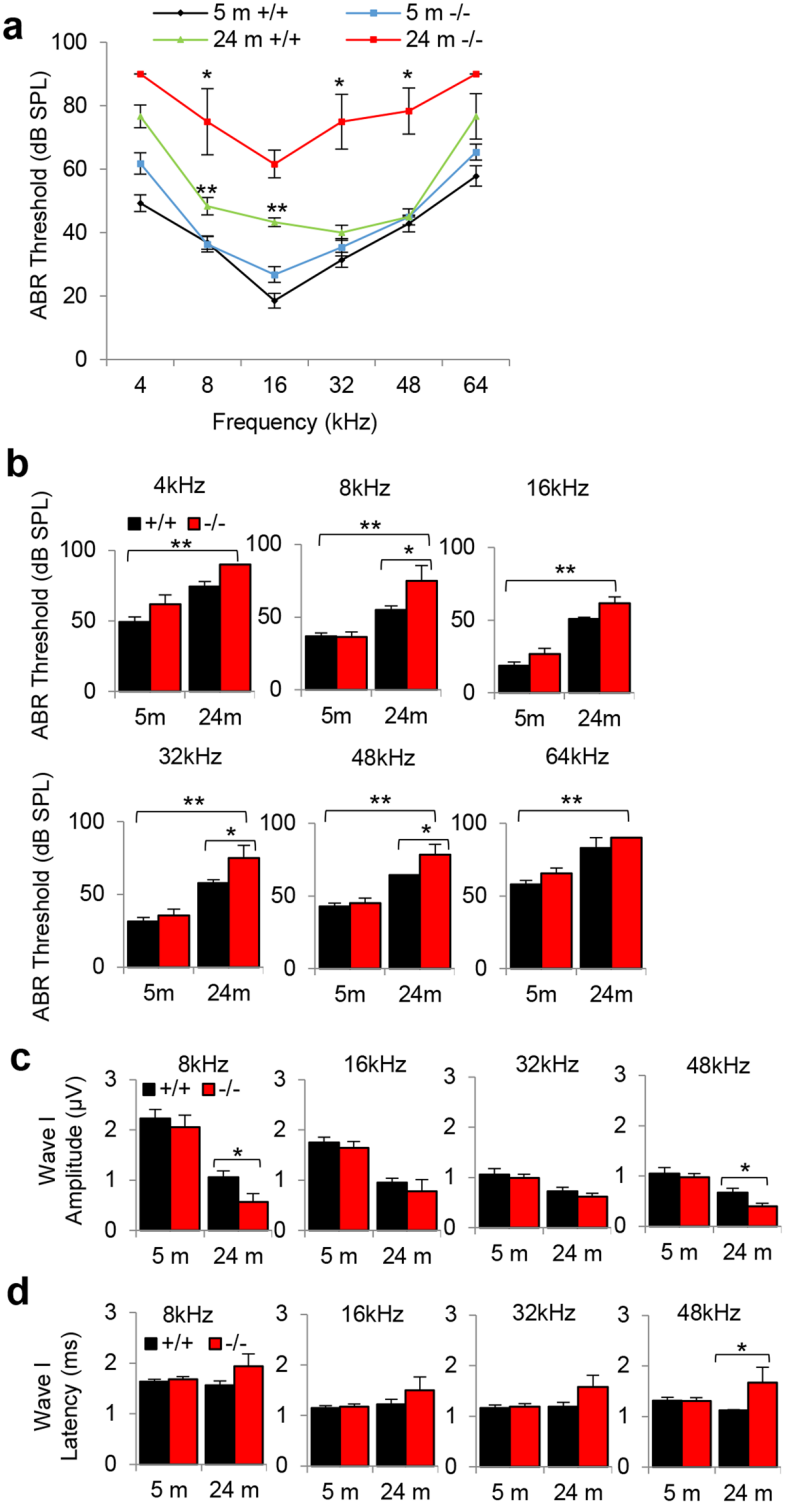
Assessment of ABR hearing thresholds, amplitudes and latencies in young and old WT and *Idh2*^−/−^ male mice. (**a**,**b**) ABR hearing thresholds were measured at 4, 8, 16, 32, 48 and 64 kHz in WT and *Idh2*^−/−^ male mice at 5 and 24 months of age (n = 3–18). (**c,d**) ABR amplitudes (**c**) and latencies (**d**) of wave I were measured at 90 dB at 8, 16, and 32 kHz in WT and *Idh2*^−*/*−^ male mice at 5 and 24 months of age (n = 3–10). Data are shown as means ± SEM. *p < 0.05 vs. middle-aged WT mice, **p < 0.05 vs. young WT.

To investigate whether loss of *Idh2* affects hearing function in young and old mice, we measured ABR (auditory brainstem response) thresholds in WT, *Idh2*^+/−^ and *Idh2*^−/−^ mice at 5 and 24 months of age. There were no differences in ABR thresholds at 4, 8, 16, 32, 48 or 64 kHz between WT and *Idh2*^−/−^ male or female mice at 5 months of age (Fig. 2a). There were also no gender differences in ABR thresholds at 4, 8, 16, 32 or 48 kHz between young WT, *Idh2*^+/−^ or *Idh2*^−/−^ mice (Fig. 2b). In mice, ABRs consist of five positive waves; wave I represents activity from the auditory nerve, while waves II–V represent activity from the central auditory system^19^. To further investigate the effects of *Idh2* deficiency on hearing function in young and old mice, ABR amplitudes and latencies for wave I were measured at 8, 16, 32 and 48 kHz at 90 dB for all animals. In agreement with ABR thresholds, there were no differences in wave I amplitudes or latencies at 8, 16, 32 or 48 kHz between 5-month-old WT and *Idh2*^−/−^male mice (Fig. 2d,e). There were also no gender differences in wave I amplitudes or latencies at all the frequencies tested in young WT or *Idh2*^−/−^ mice (Fig. 2d,e). Because there were no gender differences in ABR thresholds at all the frequencies measured between WT or *Idh2*^−/−^ mice, subsequent ABR hearing analysis at 24 months of age was conducted in male mice. Interestingly, we found that 24-month-old *Idh2*^−/−^ male mice displayed a 27–35 dB increase in ABR threshold at 8, 32 and 48 kHz compared to age-matched WT mice (Fig. 3a,b). Old *Idh2*^−/−^ male mice also displayed a 40–46% decrease in wave I amplitude at 8 and 48 kHz (Fig. 3c) and a 49% increase in wave I latency at 48 kHz (Fig. 3d) compared to age-matched WT mice. Together, these results provide direct evidence that loss of *Idh2* accelerates AHL in male mice.

### Loss of *Idh2* leads to increased oxidative DNA damage, apoptotic cell death and accelerated degeneration of hair cells and spiral ganglion neurons in the cochlea of old male mice

**Figure 4:**
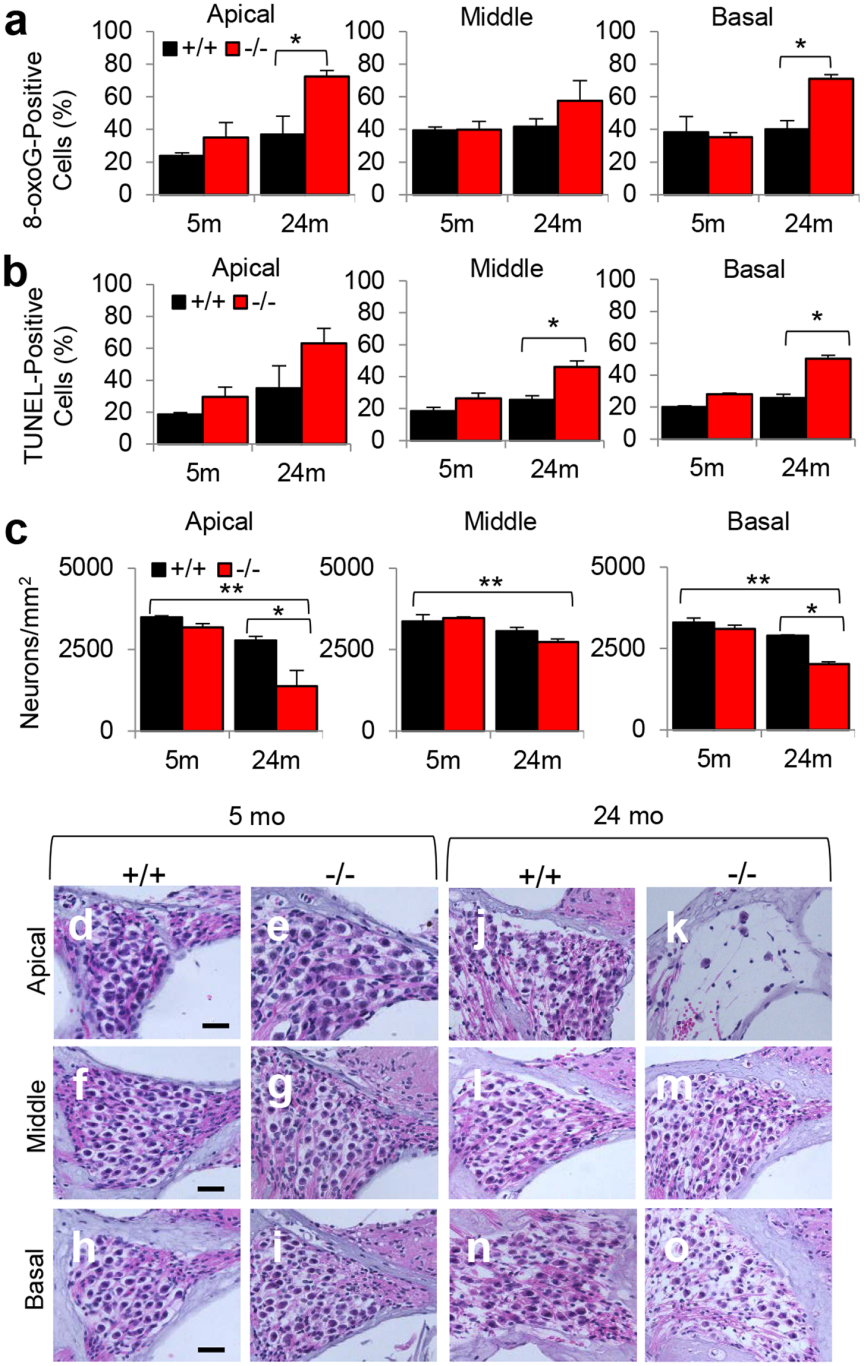
Assessment of oxidative DNA damage, DNA fragmentation, and SGN density in the cochlea of young and old WT and *Idh2*^−/−^ male mice. (**a–c**) Levels of 8-oxoguanine-positive cells (**a**) and TUNEL-positive cells (**b**) in the SGN regions, and SGN densities (**c**) were quantified in the cochlear sections from WT and *Idh2*^−/−^ male mice at 5 and 24 months of age (n = 3–4). (**d–o**) SGN regions in the apical (**d**,**e**,**j**,**k**), middle (**f,g**,**l,m**), and basal regions (**h–j**,**n,o**) of cochlear tissues from WT and *Idh2*^−/−^ male mice at 5 and 24 months of age (n = 3–4). Data are shown as means ± SEM. *p < 0.05 vs. old WT, **p < 0.05 vs. young WT. SGN = spiral ganglion neuron. Scale bar = 20 μm.

Because IDH2 is thought to be a major source of NADPH for mitochondrial glutathione and thioredoxin antioxidant defenses^9–11^, we hypothesized that loss of *Idh2* may promote oxidative DNA damage in the cochlea. To test this hypothesis, we conducted 8-oxoguanine staining in the spiral ganglion neurons (SGNs) of cochlear sections from WT and *Idh2*^−/−^ male mice at 5 and 24 months of age. There were no differences in the numbers of 8-oxoguanine-positive cells in the SGNs of apical, middle, or basal cochlear regions between 5-month-old WT and *Idh2*^−/−^ mice (Fig. 4a). However, 24-month-old *Idh2*^−/−^ mice displayed a 77% increase in 8-oxoguanine-positive SGNs in the apical and basal cochlear region compared to age-matched WT mice. It is well-documented that oxidative damage triggers cell death through apoptosis^20^. Therefore, we conducted TUNEL staining to measure nuclear DNA fragmentation, a key feature of apoptosis, in SGNs of cochlear sections from WT and *Idh2*^−/−^ male mice at 5 and 24 months of age. There were no differences in the numbers of TUNEL-positive SGNs in the apical and middle regions of the cochlea between young WT and *Idh2*^−/−^ mice (Fig. 4b). However, 24-month-old *Idh2*^−/−^ mice displayed an 82–96% increase in TUNEL-positive SGNs in the middle and basal cochlear regions.

**Figure 5:**
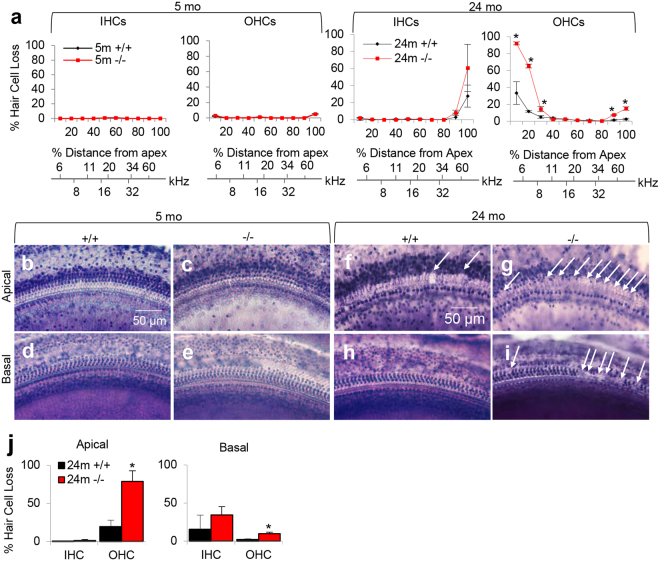
Cochleograms of young and old WT and *Idh2*^−/−^ male mice. (**a**) Cochleograms were recorded and averaged in the cochlear tissues of WT and *Idh2*^−/−^ male mice at 5 and 24 months of age (n = 4). Graphs show percent loss of IHCs and OHCs as function of percent distance from the apex (n = 4). Lower x-axes show the frequency-place map for the mouse cochlea. (**b–i**) Hair cell regions in the apical (**b,c, d,g**) and basal regions (**d,e, h,i**) of cochlear basilar membranes from 24-month-old WT and *Idh2*^−/−^ male mice. (**j**) Graphs show percent loss of IHCs and OHCs in the apical (left) and basal (right) regions in the cochlear tissues of WT and *Idh2*^−/−^ male mice at 24 months of age (n = 4). Arrows indicate missing hair cells. Data are shown as means ± SEM. *p < 0.05 vs. old WT. IHC = inner hair cell. OHC = outer hair cell. Scale bar = 50 μm.

To validate the oxidative DNA damage and apoptotic cell death results, we counted the numbers of SGNs in the apical, middle, and basal regions of the cochlea from 5-month-old and 24-month-old WT and *Idh2*^−/−^ male mice. As expected, there were no differences in the densities of SGNs in the apical, middle or basal cochlear regions between young WT and *Idh2*^−/−^ mice (Fig. 4c–i). However, 24-month-old *Idh2*^−/−^ mice displayed a 50% decrease in SGN density in the apex and a 30% decrease in SGN density in the base of the cochlea compared to age-matched WT mice (Fig. 4c, j–o). To investigate whether loss of *Idh2* leads to hair cell loss, mean cochleograms were prepared from 5- and 24-month-old WT and *Idh2*^−/−^ male mice. There were no differences in the numbers of inner hair cells (IHCs) or outer hair cells (OHCs) in the apical, middle or basal cochlear regions between young WT and *Idh2*^−/−^ mice (Fig. 5a–e). However, 24-month-old *Idh2*^−/−^ male mice displayed a 54–59% increase in OHC loss in the apical regions and a 6–13% increase in IHC loss in the basal regions of the cochlea compared to age-matched WT mice (Fig. 5a, f–j). Lastly, we measured the thickness of stria vascularis (SV) in the apical, middle, and basal regions of the cochlea from 5-month-old and 24-month-old WT and *Idh2*^−/−^ male mice. There were no differences in SV thickness in the apical, middle or basal cochlear regions between WT and *Idh2*^−/−^ mice at 5 or 24 months of age (Supplementary Fig. 4a–m). Taken together, these results provide evidence that loss of *Idh2* leads to increased oxidative DNA damage, apoptotic cell death. accelerated degeneration of HCs and SGNs in the cochlea of old male mice.

### *Idh2* deficiency results in a decline in mitochondrial NADPH redox state, mitochondrial TXNRD2 activity, cell growth and mitochondrial oxygen consumption in mouse inner ear

**Figure 6:**
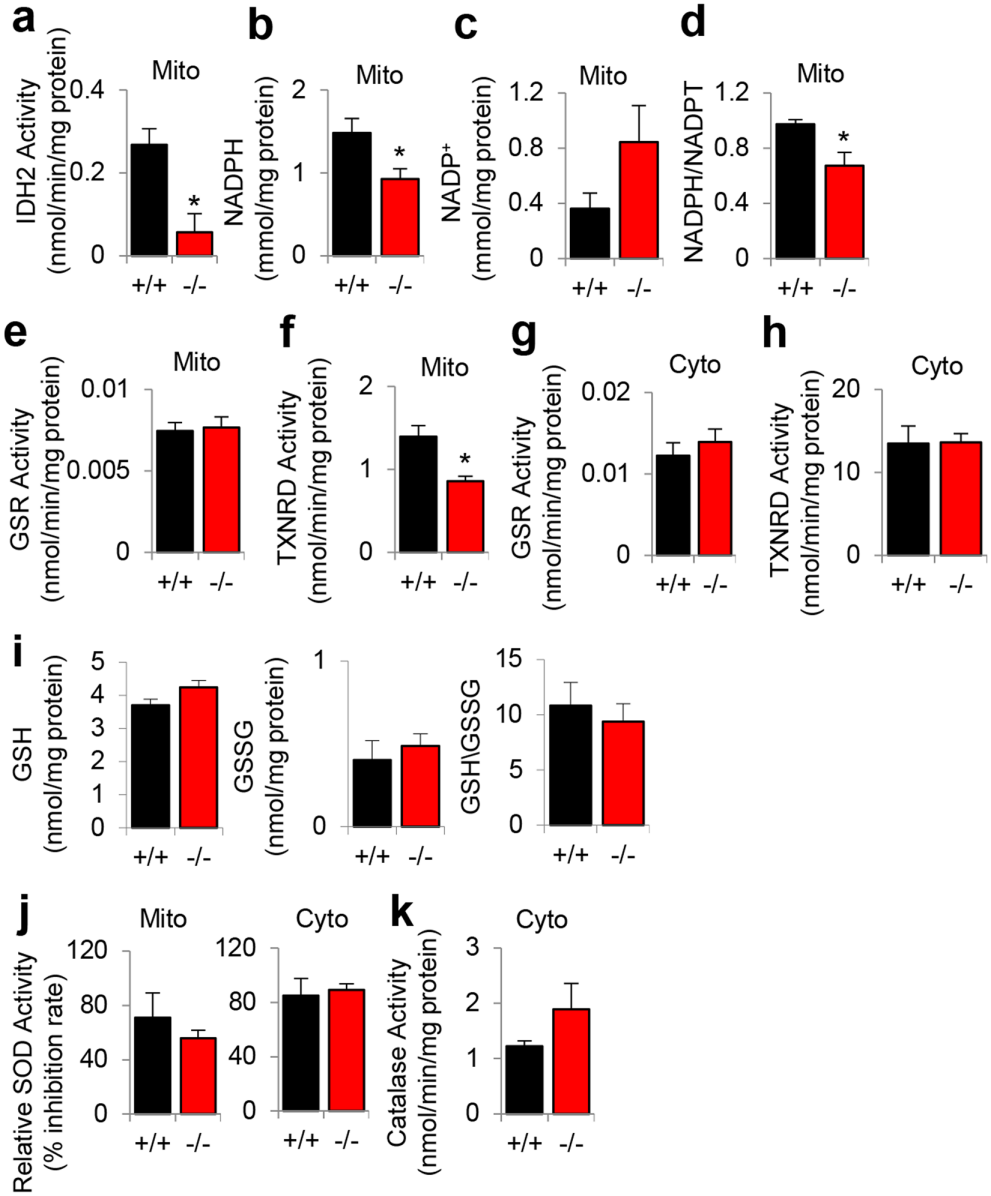
Assessment of NADPH redox state and IDH2 and antioxidant activities in the inner ear of young WT and *Idh2*^−/−^ male mice. (**a–f**) IDH2 activities (**a**), levels of NADPH (**b**), NADP^+^ (**c**), and NADPH/total NADP ratios (**d**), GSR activities (**e**), and TXNRD2 activities (**f**) were measured in the mitochondria of inner ear tissues from 5-month-old WT and *Idh2*^−/−^ male mice (n = 4). The activities of GSR (**g**) and TXNRD2 (**h**) were measured in the cytosol of inner ear tissues from 5-month-old WT and *Idh2*^−/−^ male mice (n = 4). (**i)** The levels of GSH (left), GSSG (middle), and GSH/GSSG ratios (right) were measured in the whole cell lysates of the inner ear tissues from 5-month-old WT and *Idh2*^−/−^ male mice (n = 4). (**j**,**k**) The activities of mitochondrial (left) and cytosolic (right) SOD (**j**) and cytosolic catalase (**k**) were measured in the inner ear tissues from 5-month-old WT and *Idh2*^−/−^ male mice (n = 4). Data are shown as means ± SEM. *p < 0.05 vs. WT. Mito = mitochondrial fraction, Cyto = cytosolic fraction, NADPT = total NADP, GSH = reduced glutathione, GSSG = oxidized glutathione, SOD = superoxide dismutase.

Mitochondrial GSR and TXNRD require NADPH for the regeneration of GSH and the regeneration of reduced thioredoxin, respectively^1–6^. To investigate whether loss of *Idh2* affects mitochondrial glutathione and/or thioredoxin antioxidant defenses in mouse cochlea, we measured IDH2 activities and NADPH levels in the mitochondria of inner ear tissues from 5-month-old WT and *Idh2*^−/−^ male mice. As expected, loss of *Idh2* resulted in a 78% decrease in mitochondrial IDH2 activity in the inner ear (Fig. 6a). Loss of *Idh2* also resulted in a 37% decrease in the NADPH level and a 31% decrease in the NADPH/total NADP (NADPH + NADP^+^) ratio (Fig. 6b–d). Next, we measured the activities of mitochondrial GSR and TXNRD2 in inner ear tissues from young WT and *Idh2*^−/−^ male mice. Interestingly, there were no differences in the activities of mitochondrial GSR (Fig. 6e). However, loss of *Idh2* resulted in a 38% decrease in mitochondrial TXNRD2 activity (Fig. 6f). In the cytosol, there were no differences in the activities of GSR or TXNRD1 between young WT and *Idh2*^−/−^ mice (Fig. 6g,h), while there were no differences in the ratios of GSH/GSSG in the whole cell lysates between young WT and *Idh2*^−/−^ mice (Fig. 6i). Furthermore, there were no differences in the activities of mitochondrial or cytosolic SOD (superoxide dismutase) (Fig. 6j) or cytosolic catalase (Fig. 6k) between WT and *Idh2*^−/−^ mice. Together, loss of *Idh2* results in a decline in mitochondrial NADPH redox state and TXNRD2 activity in the inner ear of young male mice.

**Figure 7:**
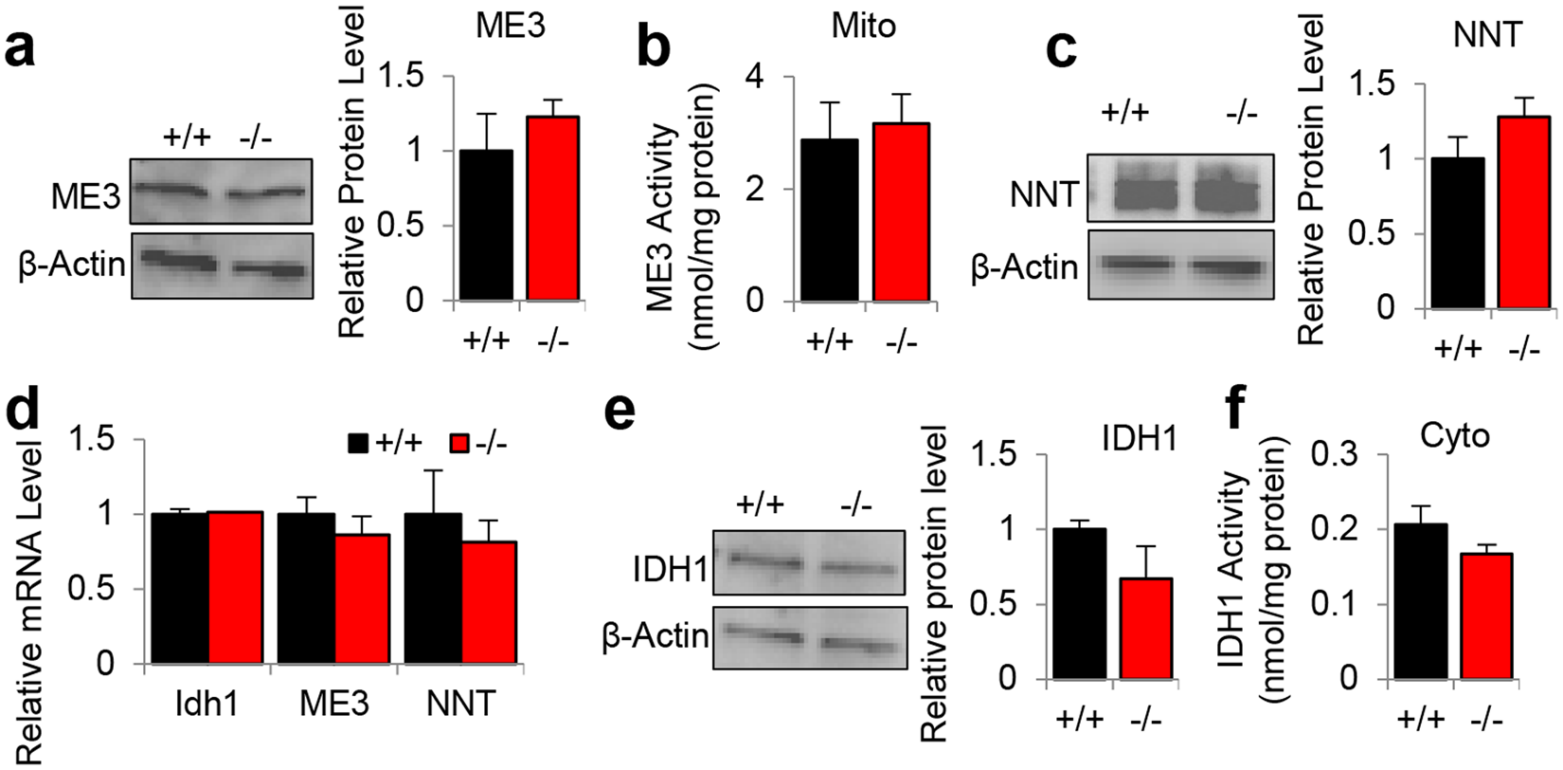
Assessment of NADPH-producing enzyme activities in the inner ear tissues from young WT and *Idh2*^−/−^ male mice. (**a–c**) Protein levels of ME3 (**a**) and the activities of ME3 (**b**) were measured in the inner ear tissues from 5-month-old WT and *Idh2*^−/−^ male mice (n = 4). The full-length blot is presented in Supplementary Fig. 5. (**c**) Protein levels of NNT were measured in the inner ear tissues from 5-month-old WT and *Idh2*^−/−^ male mice (n = 4). The full-length blot is presented in Supplementary Fig. 6. (**d**) mRNA levels of ME3, NNT and Idh1 were measured in the inner ear tissues from 5-month-old WT and *Idh2*^−/−^ male mice (n = 4). (**e**,**f)** Protein levels of Idh1 (**e**) and the activities of Idh1 (**f**) were measured in the cytosol of the inner ear tissues from 5-month-old WT and *Idh2*^−/−^ male mice. The full-length blot is presented in Supplementary Fig. 7. Data are shown as means ± SEM. Mito = mitochondrial fraction, Cyto = cytosolic fraction, ME3 = malic enzyme 3, NNT = mitochondrial transhydrogenase.

In the mitochondria, NADPH is generated by several enzymes, including NNT, ME3, and IDH2^3,7,8^. To investigate whether loss of *Idh2* affects the levels or activities of NNT or ME3 in the cochlea, we measured protein and mRNA levels of ME3 and NNT, and the activities of ME3 in the inner ears of 5-month-old WT and *Idh2*^−/−^ male mice. There were no differences in protein levels of ME3 or NNT (Fig. 7a,c, Supplementary Fig. 5 and 6), mRNA levels of *Me3* or *Nnt* (Fig. 7d), or activities of mitochondrial ME3 (Fig. 7b) in the inner ear between young WT and *Idh2*^−/−^ mice. In addition, there were no differences in mRNA levels of *Idh1*, protein levels of cytosolic IDH1 or activities of IDH1in the inner ear between WT and *Idh2*^−/−^ mice (Fig. 7e,f and Supplementary Fig. 7). Together, these results show that loss of *Idh2* does not affect the levels of NNT or ME3 in the inner ear of young male mice.

**Figure 8:**
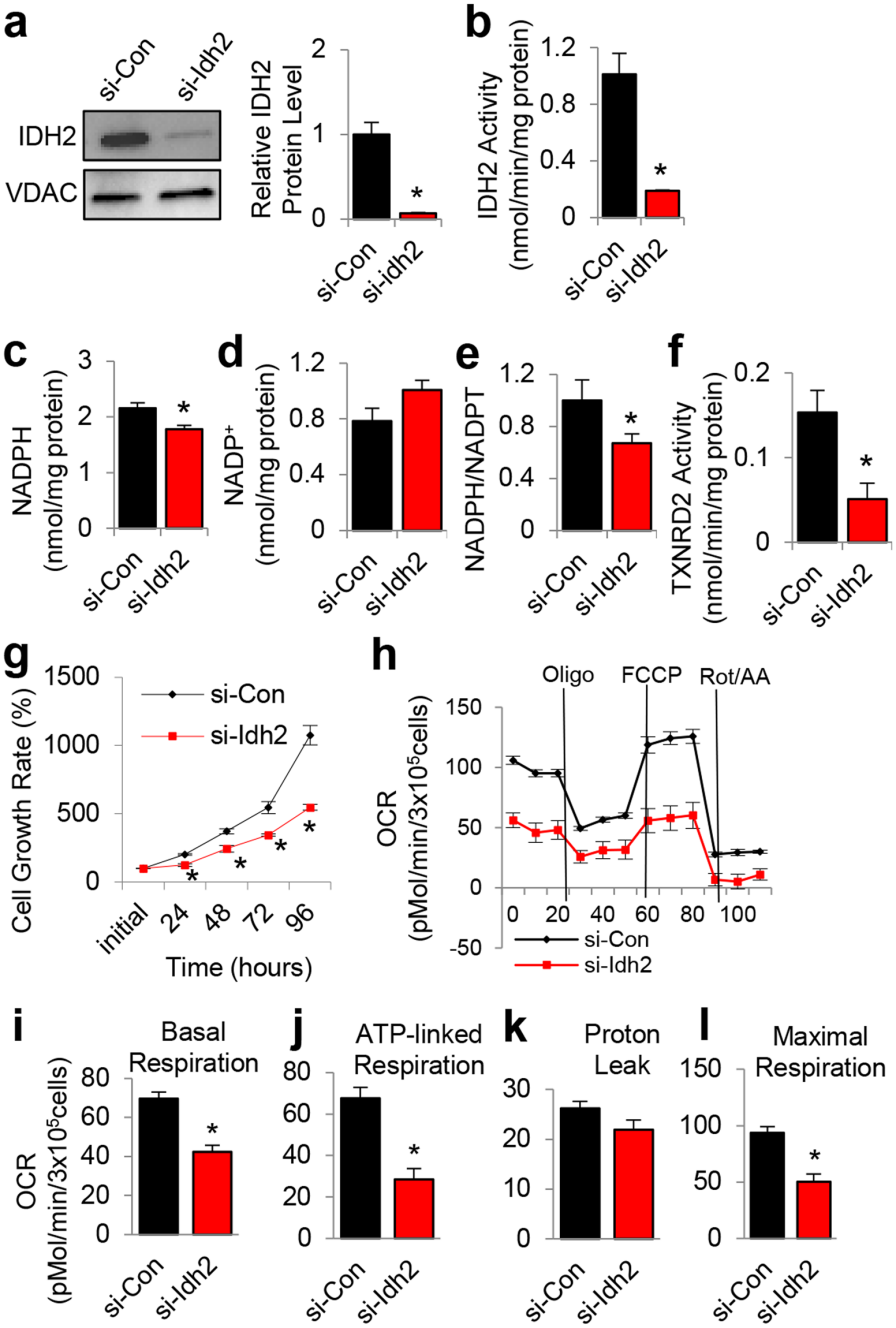
Assessment of cell growth, NADPH redox state, TXNRD2 activity and oxygen consumption rate in HEI-OC1 cell lines. (**a)** Western blotting analysis of IDH2 protein levels in the mitochondria from control and *Idh2* knockdown HEI-OC1 cells. The full-length blot is presented in Supplementary Fig. 8. VDAC was used as a mitochondrial control (n = 3). (**b–f**) IDH2 activity (**b**), NADPH (**c**) and NADP^+^ (**d**) level, and NADPH/total NADP ratio (**e**), and TXNRD2 activity (**f**) were measured in the mitochondria from control and *Idh2* knockdown HEI-OC1 cells (*n* = 3). (**g**) Cell growth rates were measured in control and *Idh2* knockdown HEI-OC1 cell lines at 0, 24, 48, 72 and 96 h (n = 3). (**h–l**) Oxygen consumption rates (OCRs) were measured in control and *Idh2* knockdown HEI-OC1 cells (n = 3). Data are shown as means ± SEM. *p < 0.05 vs. control. NADPT = total NADP, OCR = oxygen consumption rate, con = control cell line.

To validate the biochemical and histological results, we measured IDH2 activities, NADPH levels and TXNRD2 activities in the mitochondria of mouse inner ear HEI-OC1 cell lines that were transfected with siRNA targeted to *Idh2*. We confirmed that siRNA-mediated knockdown of *Idh2* resulted in a 93% decrease in IDH2 protein (Fig. 8a and Supplementary Fig. 8) and an 81% decrease in IDH2 activity (Fig. 8b). Knockdown of *Idh2* also resulted in a 33% decrease in the NADPH/total NADP ratio compared to control cells (Fig. 8c–e) and a 67% decrease in mitochondrial TXNRD2 activity compared to control cells (Fig. 8f). To investigate whether *Idh2* knockdown leads to reduced cell growth, we measured cell growth rates in control and *Idh2* knockdown cells. We found that *Idh2* knockdown resulted in reduced cell growth at 24, 48, 72 and 96 h compared to control cells (Fig. 8g). Lastly, to investigate whether *Idh2* knockdown affects mitochondrial function in HEI-OC1 cells, we measured oxygen consumption rates (OCRs) in control and *Idh2* knockdown cells. Under basal respiration conditions, *Idh2* knockdown cells significantly reduced OCRs compared to control cells (Fig. 8h,i). Under treatment with oligomycin (Oligo) which inhibits ATP synthase activity, *Idh2* knockdown cells also displayed significantly decreased ATP-linked respiration compared to control cells (Fig. 8h–j), with no significant changes in proton leak compared to control cells (Fig. 8h–k). Maximal respiration was also determined by treating cells with carbonyl cyanide-*p*-trifluormethoxyphenylhydrazone (FCCP), an uncoupling agent that disrupts ATP synthesis. Under FCCP treatment, *Idh2* knockdown cells displayed a significantly reduced OCR compared to control cells (Fig. 8h–l). Collectively, these biochemical analysis results provide evidence that IDH2 acts as the principal source of NADPH for the mitochondrial thioredoxin antioxidant defense in mouse cochlear cells.

## Discussion

Among the cellular redox couples, it is thought that the GSH/GSSG redox couple is the intracellular determinant of the antioxidant capacity because the abundance of GSH is three to four orders of magnitude higher than the other reductants^2,5,21^. In the mitochondria, IDH2 is thought to play a crucial role in protecting cells from oxidative stress by supplying NADPH to both GSR and TXNRD2^9–11^. Consistent with this view, *Idh2*-knockdown decreased levels of mitochondrial NADPH and GSH, while overexpression of *Idh2* increased mitochondrial GSH levels in NIH3T3 mouse fibroblasts^11^. In mice, homozygous mutations in *Idh2* resulted in decreased NADPH redox state, decreased glutathione redox state, and decreased ATP production in heart mitochondria^13^, while aging resulted in down-regulation of *Idh1* in the cochlea of old CBA/CaJ mice with age-related hearing loss^22^. TXNRD2 and GSR have approximately the same *K*m values for NADPH^23,24^, suggesting that the activity of GSR is likely affected by decreased NADPH level similar to that of TXNRD2. However, we found that homozygous mutations in *Idh2* resulted in decreased activity of TXNRD2, but not GSR in the mitochondria of mouse inner ears. Why did loss of IDH2 decrease the activity of TXNRD2, but not GSR? Both IDH2 and TXNRD2 are primarily or exclusively found in the mitochondrial matrix^4,5,9^. In contrast, we have shown recently that GSR is mostly present in the cytosol of mouse cochlea^21^. Importantly, NADPH is highly compartmentalized in cells and cannot diffuse between different organelles, thus maintaining different NADPH/NADP^+^ ratios to allow compartment-specific metabolic processes^9,25^. Therefore, we speculate that a loss of IDH2 and associated decline in NADPH levels may affect TXNRD2 activity, but not GSR activity in mitochondria likely because both IDH2 and TXNRD2 primarily reside in the mitochondria of mouse cochlea, while GSR is primarily localized to the cytosol

In the mitochondria, NADPH is generated from several enzymes, including NNT, ME3, and IDH2^3,7,8^, suggesting that IDH2, NNT and ME3 may be functionally redundant. However, we have demonstrated that loss of *Idh2* does not affect protein or mRNA levels of mitochondrial ME3 or NNT in the inner ear, indicating that loss of IDH2 is not compensated by ME3 or NNT for NADPH production. Mitochondria contain two isozymes for IDH: IDH2, and IDH3^3^. Both isozymes catalyze the conversion of isocitrate to α-ketoglutarate. While IDH2 converts NADP^+^ to NADPH, IDH3 converts NAD^+^ to NADH. These mitochondrial IDHs are known to act as the rate-limiting enzyme of the TCA cycle^26^. IDH2 and IDH3 not only produce NADH/NADPH, but also regulate the overall production of NADH/NADPH in the TCA cycle. Accordingly, IDH3 is thought to be essential for energy production in the TCA cycle. However, a complete loss of IDH3 activity is apparently detrimental only in the eye^27^, suggesting that IDH2, rather than IDH3, serves as the major enzyme catalyzing the oxidation of isocitrate to α-ketoglutarate in the TCA cycle in other organs. In the current study, loss of *Idh2* results in reduced levels of mitochondrial NADPH in the inner ear. This was accompanied by increased oxidative damage and increased apoptotic cell death, indicating that a complete loss of *Idh2* is detrimental in the inner ear of aged mice. Therefore, our current results and a previous study from our lab^15^, suggest that IDH2, not IDH3, regulates the overall production of mitochondrial NADPH and acts as the major enzyme catalyzing the conversion of isocitrate to α-ketoglutarate in the TCA cycle within the inner ear. Our results also suggest that IDH2 acts as the principal source of NADPH for the mitochondrial antioxidant defense in cochlea.

Large-scale sequencing studies revealed that heterozygous mutations in both cytosolic *IDH1* and mitochondrial *IDH2* are highly prevalent in World Health Organization (WHO) grade II and III astrocytomas or oligodendrogliomas and in secondary glioblastomas^9,10^. Interestingly, no mutations have been reported for IDH3 in cancer^9,28^. Subsequent studies revealed that *IDH* mutations affect a single amino acid located within the isocitrate binding site: R132 of IDH1 and the analogous R172 residue of IDH2, and the mutant IDH1 and IDH2 can convert α-ketoglutarate to the oncometabolite 2-HG and NADPH to NADP^+^^9,10,29^. This in turn causes profound metabolic and epigenetic dysregulation, including increased angiogenesis, glioma CpG island methylator phenotype (G-CIMP), and increased oxidative stress. Supporting these findings, patients with heterozygous mutations in *IDH1* or *IDH2* have highly elevated (~100- fold) amounts of 2-HG^9,10^. Interestingly, homozygous mutations in *IDH1* or *IDH2* are extremely rare in these types of brain tumors^28^, while the symptoms of brain tumors such as astrocytomas include problems with vision, speech, balance and hearing^30,31^. In the current study, we have demonstrated that a complete loss of *Idh2* results in increased oxidative damage and apoptotic cell death in the cochlea, leading to early onset AHL, in male mice. Our findings have broad implications for the roles of *IDH2* mutations in aging and cancer: First, a complete loss of IDH2 likely leads to hearing loss in older adults. We speculate that a significant reduction in mitochondrial NADPH caused by loss of IDH2 could lead to increased oxidative stress by decreasing reduced thioredoxin pools in the mitochondria of SGNs and HCs. This in turn results in decreased mitochondrial oxygen consumption, leading to age-related cochlear neurodegeneration and hearing loss. In agreement with this idea, mitochondria are a major source of ROS and a major site of ROS-induced oxidative damage and ROS-induced apoptosis^32,33^. In mice, overexpression of mitochondrial catalase resulted in a reduction in oxidative DNA damage and hair cell loss in the cochlea and delayed the onset of AHL^34^. In the current study, we have demonstrated that knockdown of *Idh2* decreased cell viability and mitochondrial respiration rates in mouse inner ear cell lines. Second, because the symptoms of brain tumors typically include problems with speech, balance and hearing^30,31^, hearing impairments associated with specific types of gliomas may be caused by *IDH2* mutations. This idea is supported by the observation that mutations in *IDH2* are highly prevalent in specific types of gliomas^9,10^. In summary, our findings provide direct evidence that loss of *Idh2* accelerates AHL, the most common form of hearing impairment^16^, in male mice. Understanding the mechanism of IDH2-mediated mitochondrial antioxidant defense may define novel therapeutic approaches for human AHL.

## Methods

### Animals

Male and female *Idh2*-deficient mice were generated by Lexicon Genetics (Woodlands, TX) and obtained from Taconic Biosciences (Hudson, NY). CBA/CaJ mice were purchased from Jackson Laboratory (Bar Harbor, ME). *Idh2*-deficient mice were backcrossed onto the CBA/CaJ background for five generations (N5). Both male and female mice were used. All animal research was conducted in accordance with the regulatory policies of and approved by the Institutional Animal Care and Use Committees of the University of Florida (UF IACUC Study #201705927).

### Genotyping and DNA sequencing

*Idh2 genotyping. Idh2*^+/−^ heterozygous males were mated with *Idh2*^+/−^ heterozygous females and their offspring were genotyped with DNA extracted from a tail clip obtained at weaning (Fig. 1a). The following primers were used for the PCR reaction:

LTR-2 5′-AAATGGCGTTACTTAAGCTAGCTTGC-3′;

TF0330-alt3 5′-GCAATACACCTGTGAAACCCTATAGG-3′;

TF0330-alt5 5′-GGCTAAGCTTTGAACTTCCTAGTTGC-3′.

The cycling parameters for PCR were as follows: one cycle at 95 °C for 2.5 min followed by 35 cycles at 60 °C for 1 min for annealing, 72 °C for 1 min for extension and 1 cycle for final extension at 72 °C for 5 min. PCR products were separated on a 1.5% agarose gel. The expected band size for the wild-type (WT) allele was 409 bps and the expected band size for the mutant (MT) allele was 238 bps (see Fig. 1a).

*Cdh23 genotyping*. Male and female *Idh2*-deficient mice were backcrossed for 5 generations onto the CBA/CaJ mouse strain that does not carry the recessive AHL-susceptibility allele (*Cdh23*^*753A*^)^17,18^. To confirm that *Idh2*^+/+^*, Idh2*^+/−^*, and Idh2*^−/−^ male and female mice have the same wild-type *Cdh23*^*753G/753G*^ genotype for *Cdh23*, we isolated DNA from these animals, amplified by PCR and then sequenced the region of DNA containing the 753^rd^ nucleotide in the *Cdh23* gene (see Fig. 1b). The following primers were used for the PCR reaction:

Cdh23-F 5′- GATCAAGACAAGACCAGACCTCTGTC-3′;

Cdh23-R 5′-GAGCTACCAGGAACAGCTTGGGCCTG-3′.

The size of the amplified PCR product was 360 bps.

### ABR hearing test

ABRs were measured with a tone burst stimulus at 4, 8, 16, 32, 48 and 64 kHz using the TDT neurophysiology workstation (Tucker-Davis Technologies, Inc., Alachua, FL) in a sound isolation booth as previously described^7^. ABR hearing thresholds were defined as the lowest level that produced a noticeable ABR response. ABR wave I amplitudes were determined by measuring the voltage difference between the highest positive value and greatest negative value for the first ABR wave as described previously^9^. Wave I latencies were measured as the amount of time elapsed from the onset of the stimulus to the peak of the first wave. We used 3–18 mice per group for ABR threshold, amplitude, and latency assessments. Following the ABR hearing measurements, tissues from the same mice were used to conduct histopathological analyses.

### Cochlear Pathology

*Histological evaluation*. After ABR hearing tests, the same animals were sacrificed by cervical dislocation. Temporal bones were excised from the head and divided into cochlear and vestibular portions as described previously^7^. Paraffin-embedded specimens of the cochlea were sliced into 5 μm sections, mounted on silane-coated slides, stained with H&E and observed under a light microscope (Leica). For histopathological analysis, we used 3–4 mice per group and examined 1–3 sections per stained slide for each mouse. In each mouse, we evaluated every third modiolar section obtained from one cochlea for a total of ten sections. Tissues from the same animals were used for SGN counting, and stria vascularis (SV) thickness measurement.

#### Cochleograms

The numbers of IHCs, first-row outer hair cells (OHC1), second-row outer hair cells (OHC2) and third-row outer hair cells (OHC3) were counted over 0.24 mm intervals along the entire length of the cochlea under a microscope at 400X magnification, as previously described^35^. The counting results were then entered into a custom computer program designed to compute a cochleogram that shows the number of missing IHC and OHC1–3 as a function of percentage distance from the apex of the cochlea. Frequency-place map for mouse cochlea was shown on the abscissa in the figures as previously described^36^.

#### SGN Counting

SGNs were counted in the apical, middle and basal regions of the cochlear sections using a 40x objective as previously described^7^. SGN density was calculated as the number of SGNs per mm^2^. Six to nine sections of the apical, middle and basal turns were evaluated in one cochlea per mouse. We used 3–5 mice per group for SGN counting.

#### Stria vascularis thickness measurements

SV thickness was measured in 40X images of H&E-stained mouse cochlear tissues as previously described^7^. Measurements were made at the basal, middle and apical regions of the cochlea for each mouse and averages of each region were calculated for each mouse. Six to nine sections of the apical, middle and basal turns were evaluated in one cochlea per mouse. We used 3–5 mice per group for SV thickness measurements.

#### Immunohistochemistry

For confocal-based immunohistochemistry, Paraffin embedded cochlea sections mounted on charged glass slides were rehydrated by standard methods and subjected to heat-based antigen retrieval in 0.01 M sodium citrate buffer pH 6.0. Sections were then washed in TBS (Tris-buffered saline) and then flooded with preincubation medium (PreInc: TBS containing 1% normal goat serum, 0.1% bovine serum albumin and 0.1% TritonX-100) for 1 h at room temperature. Primary antibodies used were: Abcam (Cambridge, MA) rabbit monoclonal antibody to IDH2 (#129180); Lifespan Bioscience (Seattle, WA) rabbit polyclonal antibody to TXNRD2 (#aa471–520); Abcam rabbit polyclonal antibody to GSR (#16801). Primary antibodies were diluted 1:50 in PreInc and sections were flooded with the diluted probes overnight at 4 °C. The next day the sections were washed 3 × 10 minutes with TBS and then flooded with FITC-conjugated goat anti rabbit IgG (Jackson Immunoresearch Laboratories, West Grove, PA) diluted 1:250 in PreInc for 3 h at 37 °C. Sections were then washed 3 × 10 min in TBS and cover slips were mounted with 60% glycerol/TBS. Immunofluorescence was observed using a Leica SP5 laser scanning confocal microscope and images were assembled into figures using Corel Draw software.

### Isolation of Mitochondria, Cytosol and Nuclei

Labyrinth tissues including bony shell, cochlear lateral wall, cochlear basilar membrane, cochlear modiolus, utricle, saccule and three semicircular canals and HEI-OC1 cells were homogenized using a tissue grinder (Wharton Dounce Tissue Grinder, Fisher Scientific) containing 1 ml of Tris buffer (10 mM Tris, 1 mM EDTA, 320 mM sucrose, pH 7.4) on ice, drawn through a 25 gauge needle (BD Biosciences, San Jose, CA) 10 times and then centrifuged at 720 g for 5 min at 4 °C to obtain a nuclear fraction (pellet). The supernatant (cytosolic and mitochondrial fractions) were transferred to a new tube and the nuclear pellet was frozen. The cytosolic and mitochondrial fractions were centrifuged at 12,000 rpm for 10 min at 4 °C. The supernatant (cytosolic fraction) was transferred to a new tube. The mitochondrial pellet was washed and re-suspended with 200 μl of 1% NP-40 buffer and centrifuged at 12,000 rpm for 10 min at 4 °C. The mitochondrial supernatant was transferred to a new tube and all fractions were stored at −80 °C.

### Western blotting

Cell lysates were centrifuged at 12,000 rpm at 4 °C for 10 min. Protein concentrations were determined using the Bio-Rad DC Assay kit. Ten to twenty micrograms of total protein were fractionated by 10% SDS-PAGE and transferred to nitrocellulose membranes (Bio-Rad, Hercules, CA). Membranes were incubated with 2% milk containing PBS, 0.05% Tween 20 (Sigma-Aldrich, St. Louis, MO) and primary antibodies overnight at 4 °C followed by horseradish peroxidase-linked secondary antibody at room temperature for 1 h. A chemiluminescent detection reagent (ECL Prime, GE Healthcare Life Sciences, Logan, UT) was used to visualize proteins. Primary antibodies for Western blotting were as follows: Idh2 (rabbit polyclonal, Proteintech, Chicago, IL), Idh1 (rabbit polyclonal, Proteintech), Nnt (mouse monoclonal antibody, Santa Cruz Biotechnology, Inc., Dallas, TX), Me3 (rabbit polyclonal antibody, GeneTex, Irvine, CA), VDAC (rabbit polyclonal, Cell Signaling, Danvers, MA) and β-actin (mouse monoclonal, Sigma-Aldrich, St. Louis, MO). A horseradish-linked rabbit or mouse secondary antibody was used. The band intensity was quantified using the ImageJ program and the levels of each protein were normalized by loading controls.

### Measurement of NADPH

NADPH levels were measured by the method of Zerez *et al*. as previously described^37^. Twenty-five µl of each unheated and heated sample was mixed with 225 µl of a reaction mixture (100 mM Tris, 5 mM EDTA, 0.5 µM thiazolyl blue tetrazolium bromide, 2 µM phenazine ethosulfate, 1.3 units glucose-6-phosphate dehydrogenase, pH 8.0) and incubated for 5 min at 37 °C. The reaction mixture was then transferred to a 96 well plate and 1 µl of 1 mM glucose-6-phosphate was added in each well to initiate the reaction. The absorbance was read at 570 nm every 10 s for 3 min in a microplate reader (Molecular Devices, Sunnyvale, CA). All samples were run in duplicate. The reaction rates were calculated and NADPH levels were determined as the ratio of NADPH (heated sample) to the total of NADP^+^ and NADPH (unheated sample).

### Isocitrate dehydrogenase activity

Activities of IDH2 and IDH1 were measured by the Kornberg method^38^. Twenty-five micrograms of the mitochondrial fraction or 20 µl of the cytosolic fraction of each sample was added to wells in a 96-well plate and then 180 µl of reaction mixture (33 mM KH_2_PO_4_∙K_2_HPO_4_, 3.3 mM MgCl_2_, 167 µM NADP^+^, and 167 µM (+)-potassium Ds-*threo*-isocitrate monobasic) was added to each well. The absorbance was immediately read at 340 nm every 10 s for 10 min in a microplate reader (Molecular Devices, Sunnyvale, CA). All samples were run in duplicate. The reaction rates were calculated and the IDH2 or IDH1 activity in the sample was defined as the production of one µM of NADPH per second.

### Malic enzyme activity

Activities of ME3 were measured as previously described^39^. Twenty-five micrograms of each sample were added to a 96-well plate with 0.1 M of Tris-HCl and 0.5 mg/mL of NADP^+^ at pH 8.0. The absorbance was immediately read and observed every 20 s for 10 min at 340 nm in a microplate reader (Molecular Devices, Sunnyvale, CA). The reaction rates were calculated and ME3 activity was defined as the production of one µM of NADPH per second.

### Antioxidant enzymatic activity

#### Glutathione reductase activity

The activity of GSR was measured in cytosolic or mitochondrial fractions using the Glutathione Reductase Assay Kit (Sigma-Aldrich, St. Louis, MO), according to the manufacturer’s instructions. The absorbance was read at 405 nm every 10 s for 2 min in a microplate reader (Molecular Devices, Sunnyvale, CA) to calculate the activity. All samples were run in duplicate.

#### Thioredoxin reductase activity

The activity of TXNRD was measured in cytosolic or mitochondrial fractions using the Thioredoxin Reductase Assay Kit (Sigma-Aldrich, St. Louis, MO), according to the manufacturer’s instructions. The absorbance was read at 412 nm every 10 s for 2 min in a microplate reader (Molecular Devices, Sunnyvale, CA) to calculate the activity. All samples were run in duplicate.

#### Catalase activity

Catalase activity was measured using the Catalase Assay Kit (Sigma-Aldrich, St. Louis, MO) in cytosolic fractions, according to the manufacturer’s instructions. The absorbance was measured at 520 nm in a microplate (Molecular Devices, Sunnyvale, CA). All samples were run in duplicate.

#### Superoxide dismutase activity

SOD activity was measured using the SOD Assay Kit (Sigma-Aldrich, St. Louis, MO) in cytosolic and mitochondrial fractions, according to the manufacturer’s instructions. The absorbance was read at 450 nm in a microplate reader (Molecular Devices, Sunnyvale, CA). The SOD activity was calculated as the inhibition rate in percent. All samples were run in duplicate.

### Measurement of total GSH and GSSG

Total GSH and GSSG were measured as previously described^40^ with modifications. Briefly, 50 µL of whole cell lysates were mixed with 2-vinylpyridine and incubated for 1 h in the dark at room temperature. Samples were then measured for total glutathione and GSSG. Samples were run in duplicate.

### Measurement of oxidative DNA damage marker

Cochlear sections on slides were rehydrated and enzymatic antigen retrieval was performed as previously described with modifications^21^. Cochlear sections were then incubated with anti-8-oxogaunine antibody (mouse monoclonal, 1:50 dilution; Abcam) to visualize 8-oxoguanine-positve cells using the M.O.M. kit (Vector Laboratories). The 8-oxoguanine-positive cells were calculated as the number of 8-oxoguanine-positive cells divided by the total number of SGNs as a percentage. We used 2–3 mice per group for 8-oxoguanine-postive cell counting.

### Measurement of apoptotic cell death marker

TUNEL staining for apoptotic nuclei was performed using a DeadEnd Colorimetric TUNEL System (Promega, Madison WI) according to the manufacturer’s instructions. Color development was accomplished with diaminobenzidine for 8 min. Duplicate sections were counterstained with hematoxylin and a cover glass was mounted to each slide with Permount mounting medium. TUNEL-positive cells were counted in the apical, middle, and basal regions of the cochlear sections using a 20 × objective as previously described^32^. The TUNEL-positive was calculated as the number of TUNEL-positive cells per mm^2^. Five sections of the unilateral apical, middle, and basal regions were evaluated in one cochlea per mouse.

### Cell culture analyses

#### Cell line

Mouse inner ear cell lines (HEI-OC1) were a gift from Dr. Federico Kalinec (Department of Head and Neck Surgery, UCLA). HEI-OC1 cells were maintained in high glucose DMEM (Life Technologies) composed of heat-inactivated 10% fetal bovine serum (HyClone FBS, GE Healthcare Life Sciences, Little Chalfont, UK) as described previously^41^.

#### Gene knockdown

To generate *Idh2* knockdown cells, HEI-OC1 cells were plated on a 6-well plate at 3 × 10^5^ cells per well the day before transfection. siRNA (Origene, Rockville, MD) targeted to mouse *Idh2* and scrambled siRNA (control) were combined with Lipofectamine RNAi Max (Life Technologies, Grand Island, NY) and were transfected to cells according to the manufacturer’s instructions.

#### Cell growth rate

After transfection, cells were incubated for 5 d. The *Idh2* knockdown cells were re-plated on a 12-well plate. Cells were allowed to grow from 24 to 96 h in DMEM. Cell growth rates were determined by the neutral red assay as previously described (Repetto *et al*., 2008). At completion of incubation, cells were incubated in DMEM with 50 µg/mL of neutral red (Sigma-Aldrich, St. Louis, MO) at 37 °C for 2–3 h. After washing, cells were then treated with 200 μl of neutral red solubilization solution (50% ethanol, 49% deionized water, and 1% glacial acetic acid (Sigma-Aldrich, St. Louis, MO) per well. The 12-well plate was incubated at room temperature on a plate shaker overnight. The OD (optical density) values of the neutral red extract in each well were measured at 540 nm in a microplate reader (Bio-Tek).

#### Oxygen consumption rate

After transfection, cells were incubated for 5 d. The *Idh2* knockdown cells were re-seeded at 30,000 cells/well onto an XF24 microplate 24 h prior to experimentation. The day before the oxygen consumption rate experiment, the sensor cartridge was calibrated using the calibration buffer (Seahorse, Agilent, Santa Clara, CA) overnight at 37 °C. One h before OCR measurement, transfected cells were washed twice with XF media and incubated in a non-CO_2_ incubator at 37 °C. Oxygen consumption rate (OCR) measurements were performed using the Mito Stress Test (Seahorse, Agilent, Santa Clara, CA), according to the manufacturer’s protocol using the Seahorse XF96 Analyzer (Agilent, Santa Clara, CA) as described previously^42^. Oligomycin, carbonyl cyanide-4-(trifluoromethoxy) phenylhydrazone (FCCP), antimycin A, and rotenone were reagents used to determine OCRs. All reagents were purchased from Sigma-Aldrich (St. Louis, MO).

### Statistical analysis

One-Way ANOVA with Tukey *post hoc* tests (GraphPad Prism 4.03) were used to analyze the ABR thresholds, wave I amplitude and latency values. Student’s t-test were used to analyze SGN densities, SV thickness, cochleograms, enzyme activities, oxidative damage markers, GSH/GSSG, NADPH, western blot analyses and cell culture analyses. All tests were 2-sided with statistical significance set at p < 0.05.

## Electronic supplementary material

Supplementary figures and figure legends
