# Supplementary material for: Loss of IDH2 Accelerates Age-related Hearing Loss in Male Mice
Source: Sci Rep. 2018 Mar 22;8:5039. doi: 10.1038/s41598-018-23436-w (PMC5864918; doi:10.1038/s41598-018-23436-w)
Supplement: Supplementary file 1 — Supplementary figures and figure legends [file 41598_2018_23436_MOESM1_ESM.pdf]

Supplementary Information for

Loss of IDH2 Accelerates Age-related Hearing Loss in Male Mice

Karessa White,[1] Mi-Jung Kim,[1] Chul Han,[1] Hyo-Jin Park,[1] Dalian Ding,[2] Kevin Boyd,[1] Logan Walker,[1] Paul Linser,[3] Zaimary Meneses,[1] Cole Slade,[1] Jonathan Hirst,[1] Katherine Santostefano,[4] Naohiro Terada,[4] Takuya Miyakawa,[5] Masaru Tanokura,[4] Richard Salvi,[2] Shinichi Someya[1]

Affiliations:

[1]Department of Aging and Geriatric Research, University of Florida, Gainesville, Florida 32610.

[2]Center for Hearing and Deafness, State University of New York at Buffalo, New York 14214.

[3]Whitney Laboratory, University of Florida, St Augustine, Florida 32080. [4]Department of Pathology, Immunology and Laboratory Medicine, University of Florida, Gainesville, FL 32610.

[5]Department of Applied Biological Chemistry, University of Tokyo, Yayoi, Tokyo, 113.

Correspondence and requests for materials should be addressed to S.S. (someya@ufl.edu)

Consents

Supplementary Figure 1. Full-length gel image.

Supplementary Figure 2: Body weight.

Supplementary Figure 3. Full-length blot image.

Supplementary Figure 4: Assessment of stria vascularis thickness in the cochlea of young and old WT and *Idh2*$^{-/-}$ mice.

Supplementary Figure 5. Full-length blot image.

Supplementary Figure 6. Full-length blot image.

Supplementary Figure 7. Full-length blot image.

Supplementary Figure 8. Full-length blot image.

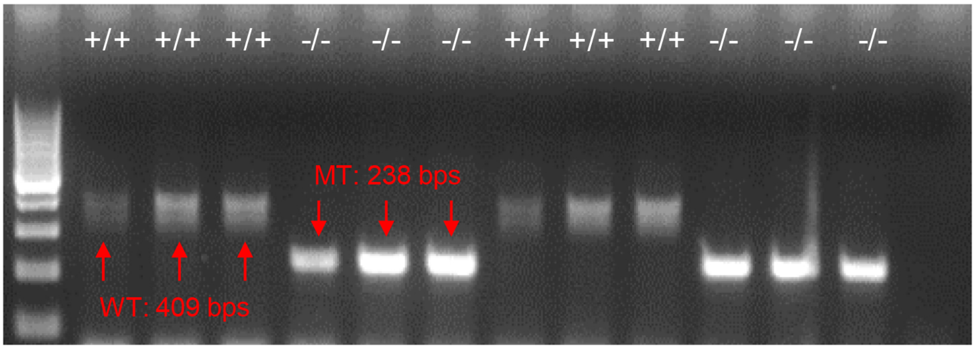

Supplementary Figure 1. Full-length gel image.
The full-length gel image of Figure 1a. Arrow indicate the cropped image region of Figure 1a.

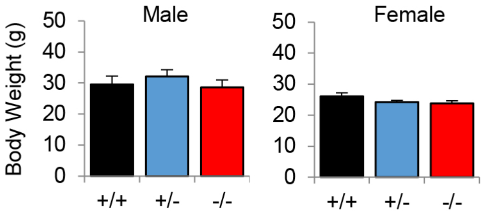

Supplementary Figure 2. Body weight.
Body weight was measured in young WT and *Idh2*$^{-/-}$ male (left) and female (right) mice at 5 months of age (n = 10). Data are shown as means ± SEM.

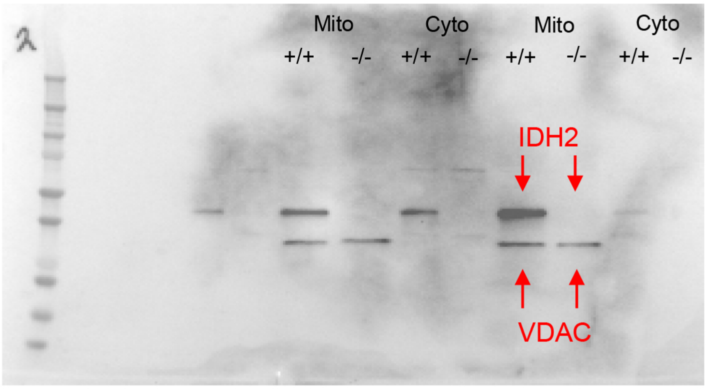

Supplementary Figure 3. Full-length blot image.
The full-length blot image of Figure 1c. Arrows indicate the cropped image regions of Figure 1c.

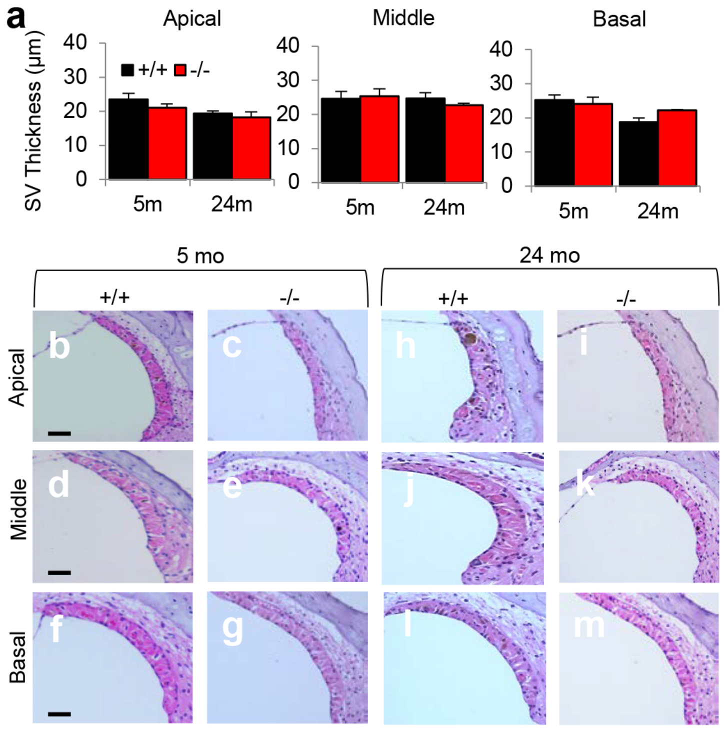

Supplementary Figure 4. Assessment of stria vascularis thickness in the cochlea of young and old WT and *Idh2*$^{-/-}$ mice.

(**a**) The thickness of SVs was measured in the apical, middle and basal regions in the cochlea of WT and *Idh2*$^{-/-}$ mice at 5 and 24 months of age (n = 4). (**b-m**) SV regions in the apical, middle and basal regions of cochlear tissues from WT and *Idh2*$^{-/-}$ male mice at 5 (**b-g**) and 24 (**h-m**) months of age (n = 4). Data are shown as means ±SEM. SV=stria vascularis. Scale bar = 20 µm.

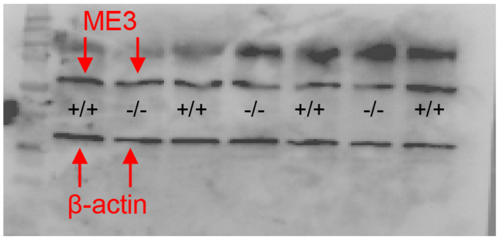

Supplementary Figure 5. Full-length blot image.
The full-length blot image of Figure 7a. Arrows indicate the cropped image regions of Figure 7a.

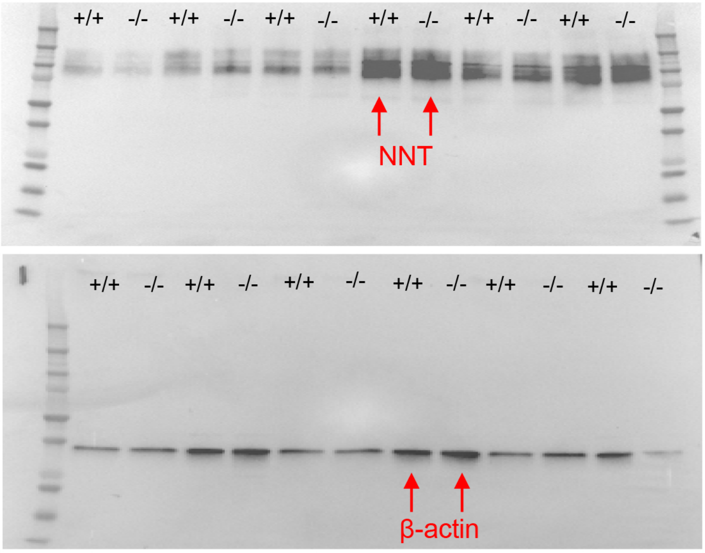

Supplementary Figure 6. Full-length blot image.
The full-length blot images of Figure 7c. Arrows indicate the cropped image regions of Figure 7c.

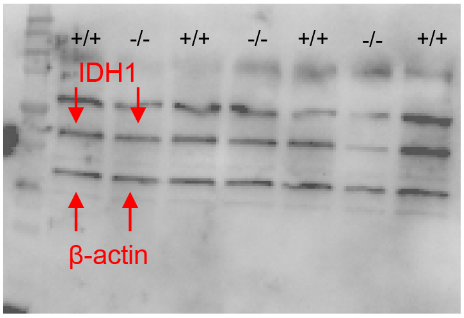

Supplementary Figure 7. Full-length blot image.
The full-length blot image of Figure 7e. Arrows indicate the cropped image regions of Figure 7e.

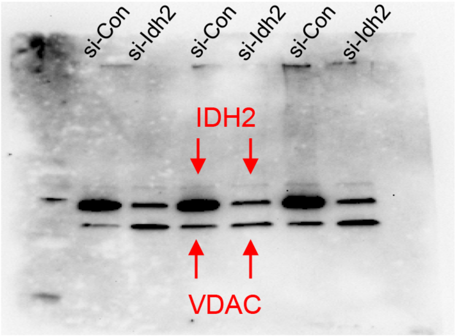

Supplementary Figure 8. Full-length blot image.
The full-length blot image of Figure 8a. Arrows indicate the cropped image regions of Figure 8a.
